# Reviewed article: Lopes BT, Oliveira CH, Souza PAA, Cordeiro-de-Barros RM, Teixeira TJ, Santos RM, Menezes AS, Abreu FVS. Ocurrence of Nyssomyia intermedia (Diptera: Psychodidae) in intradomiciliary environments: a potential vector of american cutaneous leishmaniasis in Montezuma, 2024. Epidemiol Serv Saude. 2025:34;e20250198.

**DOI:** 10.1590/S2237-96222025v34e20250198.b

**Published:** 2025-10-27

**Authors:** Tiago Silva da Costa

**Affiliations:** 1 Universidade Federal do Amapá, Macapá, AP, Brazil

## Peer review B

## Questionnaire

Does the manuscript contain new and significant information to justify publication? Yes

Does the Abstract (Summary) clearly and accurately describe the content of the article? Yes

Is the problem significant and concisely stated? Yes

Are the methods described comprehensively? Yes

Are the interpretations and conclusions justified by the results? Yes

Is adequate reference made to other work in the field? No

## Manuscript Structure

Length of article is: Adequate

Number of tables is: Adequate

Number of figures is: Too few

Please state any conflict(s) of interest that you have in relation to the review of this paper. None

Interest: Excellent

Quality: Good

Originality: Average

Overall: Good

Recommendation: Minor Revision

## Comments

O artigo traz contribuições importantes para a compreensão do papel de Nyssomyia intermedia na transmissão da leishmaniose tegumentar americana no intradomicílio, especialmente em áreas rurais do Norte de Minas Gerais. No entanto, seria interessante uma discussão mais ampla, já que o artigo faz referência a estudos anteriores sobre Nyssomyia intermedia , mas incluir uma comparação mais detalhada com outros trabalhos realizados em regiões semelhantes (mesmo bioma ou condições socioeconômicas) ampliaria a relevância e a utilidade do trabalho para a comunidade científica e para o sistema de vigilância epidemiológica.

